# Imaging Findings From Different Pathological Types of Oral and Maxillofacial Intramuscular Hemangiomas for Selecting Optimum Management

**DOI:** 10.3389/fonc.2021.792554

**Published:** 2022-01-31

**Authors:** Dan Zhu, Xiaoqing Dai, Jingbo Wang, Chunye Zhang, Xiaofeng Tao, Lizhong Wu, Ling Zhu

**Affiliations:** ^1^ Department of Radiology, Shanghai Ninth People’s Hospital, Shanghai JiaoTong University School of Medicine, Shanghai, China; ^2^ Department of Pathology, Shanghai Ninth People’s Hospital, Shanghai JiaoTong University School of Medicine, Shanghai, China

**Keywords:** intramuscular hemangioma, magnetic resonance image, computed tomography, histopathological classification, blood supply status

## Abstract

**Objectives:**

To assess computed tomography (CT) and magnetic resonance imaging (MRI) findings of intramuscular hemangiomas (IMHs) in oral and maxillofacial region and correlate them with the histopathological classifications for selecting optimum management.

**Methods:**

The clinical data and pretreatment findings of 32 patients with pathologically proven IMHs on CT (n = 10), MRI (n = 27), or both (n = 5) were analyzed retrospectively. Correspondence of clinical and imaging characters with 3 different pathological classifications (cavernous, capillary, and mixed) of IMHs was studied. A number of pitfalls and overlap of imaging features can result in misdiagnosis of different IMHs lesions.

**Results:**

Four patients had multi-muscular lesions, and 28 had single-muscular lesions. The predilection site were the tongue (11 cases, 34.4%) and the masseter muscle (10 cases, 31.2%). Cavernous type (17 cases, 53.1%) was the most common IMHs type. All patients showed slightly hypointense or isointense on T1-weighted imaging, 3 patients showed hyperintense on T2-weighted imaging and the others showed slightly hyperintense. The most common enhancement pattern was progressive (29 cases, 90.6%). The capillary type (9 cases, 28.1%) and mixed type (6 cases, 28.1%) of IMHs on imaging indicated characteristics of lesions with rich blood supply status, the cavernous type (17cases, 53.1%) of IMHs belonged to relatively poor blood supply lesions. A total of 5 patients (15.6%) were initially misdiagnosed, there were recurrences in 4 IMHs patients. Extra functional MRI (fMRI) was performed on these 5 misdiagnosed patients, the average ADC of the 5 patients was 1.50 × 10^−3^ mm^2^/s. The presence of vermicular vessels was different among these three types of IMHs.

**Conclusions:**

The reason for the misdiagnosis in localized IMHs may be the obvious border of mass-like lesions and the lack of enlarged vessels. Combined evaluation of presence of vermicular vessels and fMRI might be more accurately for determining the IMHs and create a preoperative plan.

## Introduction

Intramuscular hemangiomas (IMHs) are unique vascular malformations that are benign and most commonly occur in the trunk and extremities. In previous study, only 0.8% of hemangiomas were located in the muscle (1, 2). Of the reported cases, approximately 45% are located in the lower extremities (3). In general, about 14% of IMHs are originated from the muscles of the oral and maxillofacial region (4). In 1982, Mulliken et al. classified vascular lesions based on clinical observation and histopathology into hemangiomas and vascular malformations (5). Hemangiomas that feature with endothelial cell proliferation are true tumors, whereas vascular malformation is not since it simply manifests as abnormal vasculatures. Current opinions suggest that IMHs is more close to a vascular malformation rather than a hemangioma (6). However, IMHs can often be accompanied by endothelial cell proliferation. In 1972, Allen and Enzinger proposed the currently well-known categorization, which divides IMHs into three types based on the size of vessel diameter, namely, venous (diameter >140 μm), capillary (diameter <140 μm), and mixed type (7). Different sizes of blood vessels in the lesions develop different blood supplies, which can be roughly classified into 2 types, the poor (smaller volume and slower blood flow in the lesions) and the rich blood circulation type (larger volume and faster blood flow in the lesions).

IMHs are usually asymptomatic, inconspicuous, and difficult to detect in the early stages. When the tumor grows larger, it may compress and push adjacent nerves and muscles, causing various symptoms. It is reported in the literature that the preoperative diagnosis rate is only 8 to 19% (8). IMHs are often misdiagnosed because of the variable blood supply and uncertain symptoms (9). For IMHs with a rich blood supply, arterial embolization before surgery would be needed to reduce intraoperative bleeding; however, for IMHs with a poor blood supply, simple embolization would be sufficient enough before surgery (10). In addition, IMHs become more difficult to treat as they enlarge.

Evaluation of blood supply of IMHs through preoperative imaging provides practical information for the selection of treatment methods. Different pathological types of IMHs express distinct images. Among all of the publications, results of computed tomography (CT) and magnetic resonance imaging (MRI) depicted IMHs as venous malformations since the non-contrast CT scanning density of the lesions was relatively lower than surrounded muscles and the enhancement are not clear (poor blood circulation type) (4). Meanwhile, a few studies have also shown that IMHs lesions were obviously enhanced in contrast CT and MRI (rich blood circulation type) (11, 12). Currently no study has been proposed to identify the relationship between the 3 pathological types of IMHs and the blood supply status. In addition, due to the various blood supply status, IMHs clinically may be confused with malignant tumors (9). Therefore, familiarity with IMHs imaging features and the preoperative evaluation of the blood supply status are of great significance for the precise diagnosis and treatment of IMHs.

## Materials and Methods

Medical records of 32 patients with oral and maxillofacial IMHs from January 2001 to December 2020 (average age: 36.28 ± 14.49 years), namely, 7 males (21.8%) and 25 females (78.1%), were analyzed in this study. Before surgery, 10 patients received contrast CT scanning, 27 patients conducted contrast MRI scanning, 5 patients received both scanning.

CT scanning was performed with Aquillion ONE (Canon Medical Systems, Otawara, Japan) or Brilliance iCT (Philips Healthcare, Cleveland, OH, USA) with the following parameters: tube voltage of 120 kV/250 mAs, slice thickness of 5 mm, non-contrast scanning, and contrast scanning. CT images with contrast were 1.25-mm reconstructed using Philips Extended Brilliance Workspace software. A nonionic contrast agent (350 mg I/ml) was intravenously injected at a rate of 1.5–2.0 ml/s.

MRI scanning were performed on a GE 750 3.0-T or 1.5-T Sigma Twin Speed MR scanner (General Electric Medical Systems, Milwaukee, WI, U.S.) with a head and neck array coil. In the beginning, subjects were examined by conventional MRI scanning. Scanning sequences and parameters were described as follows: 1) Fast recovery fast spin echo T2WI (FRFSE T2WI) with repetition time (TR) of 4,020–4,820 ms, echo time (TE) of 84.8 ms, for 3 times; 2) Fast spin echo T1WI (FSE T1WI) with TR of 400–600 ms, TE of 8–9.9 ms, for 2 times. 3) Diffusion-weighted imaging (DWI) with using a single-shot spin-echo echoplanar imaging sequence (TR/TE, 2,200/70 ms; matrix, 128 × 128 mm; FOV, 240 × 240 mm; and b = 1,000 s/mm^2^). ADC maps were generated. 4) a single-voxel 1H-MRS localizer scan was first performed, which involved a single or multichannel surface head coil for T1W or T2W spin-echo sequence transverse scans (T1W: TR/TE, 460/7.9 ms; T2W: TR/TE, 4,700/85 ms).

Dynamic contrast enhanced (DCE)-MRI was performed using a gradient recalled echo–echo planar imaging (GRE) with TR of 400–600 ms, TE of 58–9.9 ms, thickness of 5 mm, gap of 0 mm, FOV of 24–26 cm, and matrix of 256 × 192 mm. The gradient strength was set as 30 Mt/m. The MRI contrast agent, gadopentetic acid (Gd-DPTA), was injected at a rate of 2.5 ml/s and a dose of 0.2 ml/kg. A single scan took 5 s, and the total scan time was 220–280 s.

The section with the maximum diameter of lesions was selected as the central section. The mean lesion ADC was evaluated by manually drawing an ROI on the b = 1,000 diffusion images and copying this ROI to the corresponding ADC map by two radiologists, who had more than 3 years of experience in the MRI of head and neck lesions and were blinded to the clinical outcomes. The ROI of ^1^H-MRS was set according to the location and size of the lesion, and a prescan was performed on the ROI, which included receiver gain transference, water center frequency adjustment, ROI shimming, and water suppression. The size and location of the ROIs in multiple ^1^H-MRS images were held constant within the same lesion. This procedure was followed by point-resolved spectroscopy (PRESS) (TR/TE = 1,500/144 ms) and eight excitations and a ^1^H-MRS spatial localizer scan with a water linewidth <15 Hz. Metabolites, which included choline (Cho), creatine (Cr), inositol (MI), water, and N-acetyl-aspartate (NAA), were automatically integrated using PROBE-SV software. A positive result was defined as the detection of a Cho peak (3.2 ppm) in ^1^H-MRS; a negative result was defined as no detection of a Cho peak.

Clinical variables including gender, age, location of lesions, treatment, recurrence and imaging variables such as the visibility of phleboliths or vermicular vessels, the lesion morphology on MRI, and MRI signal characteristics of each IMHs patients were collected and summarized to evaluate the correlation with pathological types.

## Results

### Clinical and Pathological Findings

**Figure 1:**
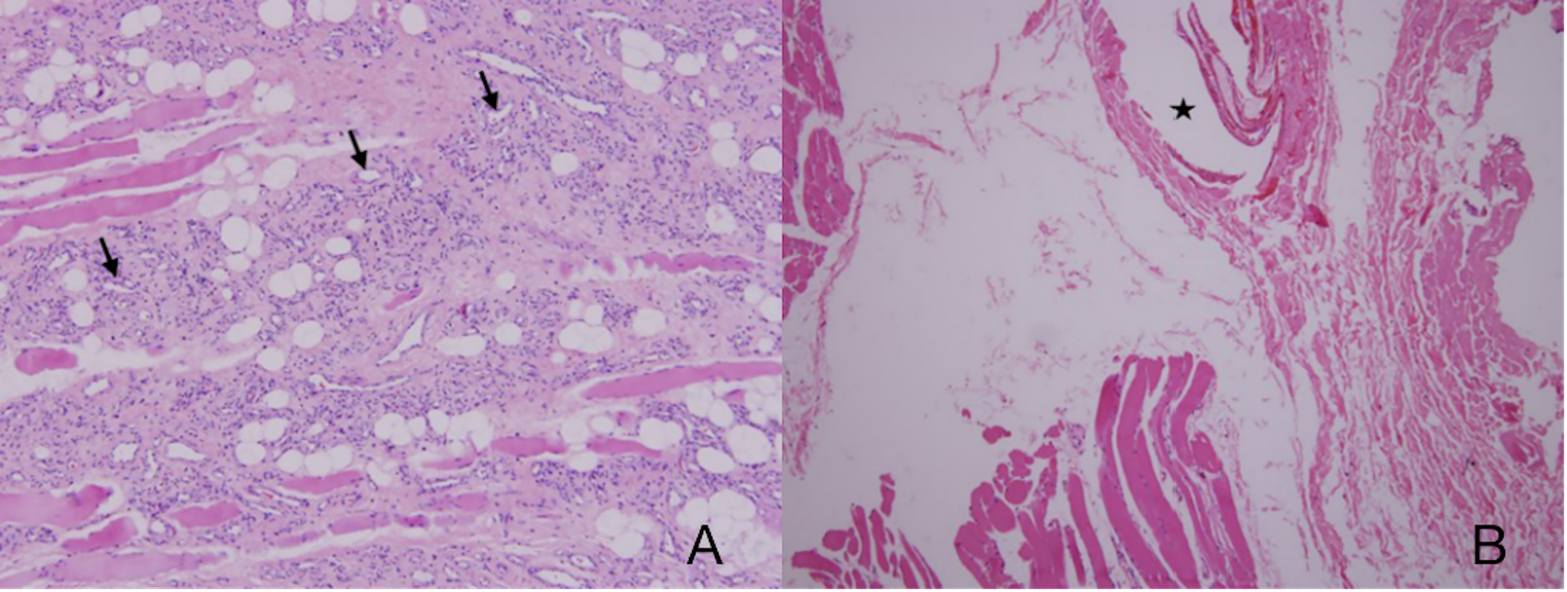
**(A)** Capillary-type IMHs: diameter <140 μm (arrows). **(B)** Cavernous-type IMHs: blood vessel diameter >140 μm (star). Hematoxylin and eosin (H&E) staining. Original magnifications, ×100.

A total of 32 patients were pathologically confirmed as IMHs cases (**Table 1** and **Supplementary Table 1**), namely, 3 with lesions in the lip (9.4%), 11 in the tongue (34.4%), 10 in the masseter (31.2%), 3 in the temporal muscle (9.4%), 4 in multiple muscles (12.5%), and 1 in skull base (3.1%). Among all, 4 patients (12.5%) received embolization prior to surgical resection, and 28 patients (87.5%) underwent direct surgical resection without embolization. Histopathological findings categorized 9 cases into the capillary type (28.1%) (**Figure 1A**), 6 cases into the mixed type (18.8%), and 17 cases into the cavernous type (53.1%) (**Figure 1B**).

**Table 1:** Clinical and pathological characters of all 32 patients with intramuscular hemangiomas.

Parameter	n (%)
Age (years)	36.3±14.5
Sex	
Male/female	7:25
Sites	
Single muscular localized	27 (84.4%)
Multiple muscular localized	5 (15.6%)
Pathological diagnosis	
Cavernous	17 (53.1%)
Mixed	6 (18.8%)
Capillary	9 (8.3%)
Recurrence	4 (12.5%)
Misdiagnosis	5 (15.6%)
Phlebolith	10 (31.2%)
Vermicular vessels	10 (31.2%)
Hemorrhage or Hemosiderin	2 (6.2%)
Thrombosis	5 (15.6%)
Teatment	
Resection	28 (87.5%)
TAE+resection	4 (12.5%)

In some of 32 patients, the diagnosis of IMH was not considered at initial radiologic evaluation. The misdiagnosed cases considered are summarized in **Table 1**. The lesion was too small to be displayed clearly (6.3%), and it was impossible to make an imaging diagnosis. Some lesions were misdiagnosed as granulomatous lesions (1 case, 3.1%) or sarcomas (4 cases, 12.5%). There were recurrences in 4 IMH patients: the mixed type (1 case, 3.1%) and the cavernous type (3 cases, 9.3%). The median follow-up time was (41.52 ± 24.59) months. The percentage of having vermicular vessels was apparently different among the 3 IMHs types, which were 17.6, 33.3, and 66.7% in cavernous IMHs, capillary IMHs, and mixed IMHs cases, respectively.

### Radiologic Findings

**Tables 2** and **3** display the size and morphology of IMHs (**Supplementary Tables 2, 3**). According to the radiologic images of 32 cases, patients can be divided into the single muscle localized type (n = 28, 87.5%) and multiple muscle diffuse type (n = 4, 12.5%). The mean diameter of the dominant lesion was 2.9 cm with a range of 1.1–7.5 cm. All the lesions were lobulated (n = 1), irregular (n = 27), or oval (n = 4). The lobulated or irregular lesions were much larger than the oval lesions. All the lesions were typically well-circumscribed.

**Table 2:** Preoperative CT findings of 10 patients with intramuscular hemangiomas.

Parameter	n(%)
Sites	
Single muscular localized	8 (80%)
Tongue	1 (10%)
Parotid region	1 (10%)
Lip	3 (30%)
Masseter	2 (20%)
Buccal region	1 (10%)
Multiple muscular localized	2 (20%)
Diameter (cm)^#^	3.24±2.12
Shape	
Irregular	9 (90%)
Oval	1 (10%)
Hemorrhage or Hemosiderin	0
Phlebolith	4 (40%)
Vermicular vessels	7 (70%)
Enhancement Pattern	
Progressive	7 (70%)
Quick wash-in, slow washout	2 (20%)
NA	1 (10%)
Pathological diagnosis	
Cavernous	4 (40%)
Mixed	4 (40%)
Capillary	2 (20%)

NA, not available. ^#^For multiple lesions, we selected the largest diameter of the largest lesion; for single lesions, we selected the largest diameter of the lesion.

**Table 3:** Preoperative MRI findings of 27 patients with intramuscular hemangiomas.

Parameter	n (%)
Sites	
Single muscular localized	24 (88.9%)
Tongue	11 (40.7%)
Skull base	1 (3.7%)
Lip	1 (3.7%)
Masseter	9 (33.3%)
Buccal region	2 (7.4%)
Multiple muscular localized	3 (11.1%)
Diameter (cm)^#^	2.91±1.46
Shape	
Irregular	22 (81.5%)
Lobulated	1 (3.7%)
Oval	4 (14.8%)
T1-Weighted MRI	
Hypointense	8 (29.6%)
Hypointense and patchy hyperintense	1 (3.7%)
Isointense	3 (11.1%)
Slightly hypointense	15 (55.5%)
T2-Weighted MRI	
Hyperintense	2 (7.4%)
Hyperintense and few striped hypointense	1 (3.7%)
Slightly hyperintense	23 (85.1%)
Slightly hyperintense and few striped hypointense	1 (3.7%)
Phlebolith	7 (25.9%)
Hemorrhage or Hemosiderin	2 (7.4%)
Vermicular vessels	6 (22.2%)
Enhancement Pattern	
Progressive	22 (81.5%)
Quick wash-in, slow washout	3 (11.1%)
NA	2 (7.4%)
Pathological diagnosis	
Cavernous	15 (55.5%)
Mixed	4 (14.8%)
Capillary	8 (29.6%)

NA, not available. ^#^For multiple lesions, we selected the largest diameter of the largest lesion; for single lesions, we selected the largest diameter of the lesion.

**Figure 2:**
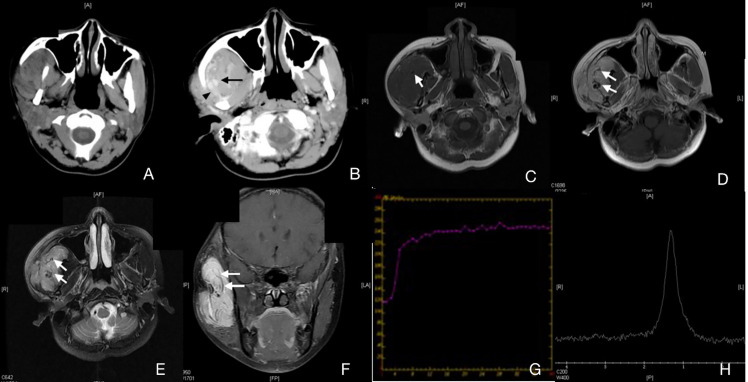
Case 4 (**Supplementary Table 1**), capillary IMHs. **(A)** Axial CT image showing an enlarged right masseter with uneven attenuation. **(B)** Axial enhanced CT image showing an apparently enhanced vermicular vessel (arrow) and an enlarged regurgitant vein (arrowhead). **(C)** Axial T1WI showing a lesion with an intermediate signal intensity and a vague border. **(D)** Axial T2WI showing a lesion with a slightly high signal intensity and a blurred border. **(E)** Axial enhanced fat suppressed T1WI showing an unevenly enhanced lesion in the right masseter. **(F)** Coronal enhanced fat suppressed T1WI showing a diffuse, irregular lesion with low signal intensity and a striped structure. This structure can also be found in panels **(C–E)**. **(G)** TIC analysis showed quick wash-in and slow washout. **(H)** MRS showing no Cho peak at 3.2 ppm.

Unenhanced CT findings—10 patients had preoperative CT scans, and the CT findings are shown in **Table 2**. These lesions commonly showed uneven attenuation on unenhanced CT (**Figure 2A**). There was no obvious hemorrhage or hemosiderin on CT in 10 patients. Phlebolith was found in 4 patients (40%), and vermicular vessels in 7 patients (70%).

Unenhanced MRI findings—The preoperative MRI findings in 27 patients are shown in **Table 3**. A total of 12 cases (37.5%) presented with homogeneous masses, which were no obvious to be found phleboliths, vermicular vessels, or bleeding/hemosiderin. These lesions were slightly hypointense or isointense on T1-weighted imaging and slightly hyperintense on T2-weighted imaging in comparison with the muscle. Phleboliths were found in 7 cases (25.9%), and vermicular vessels in 6 patients (3.7%). There were some obvious hemorrhage or hemosiderin on MRI in 2 patients (7.4%). No significant decrease in signal intensity was observed in the fat-saturation sequence in all cases.

Characteristics and patterns of enhancement—In all cases, contrast media was applied. In the enhanced CT examination, the enhancement pattern of 7 patients (70%) was progressive, and 2 patients (20%) presented with quick wash-in and slow washout. In the enhanced MRI examination, the enhancement pattern of 22 patients (81.5%) was progressive, and 3 patients (11.1%) presented with quick wash-in and slow washout. In terms of pathological types, all the cavernous types were progressive. In the capillary type, 2 cases (22.2%) were quick wash-in and slow washout, while 1 case (16.7%) mixed type was in the same enhancement pattern. These findings suggested that the blood supply status might be an imaging feature of this disease, which was also confirmed in pathologic examinations.

Pathological types and the blood supply status—Expression of capillary and mixed type of IMHs were similar on CT images. In the non-contrast images, muscle tissues in the lesion area were obviously enlarged, boundaries were unclear, and the density was similar to that of normal muscles (**Figure 2A**). Signals were strongly intensified with contrast. An enhanced streak-like structures passing through the muscles can be observed, which was considered as lumen of lesions or blood supply arteries. Enlarged venous reflux vessels around the lesions were also observed (**Figure 2B**). These characteristics were consistent with lesions with rich blood supply status. Results of the capillary type and mixed type of IMHs on MRI indicated characteristics of lesions with rich blood supply status as well, with irregular intermediate signal intensity on T1WI and slightly high signal intensity on T2WI, both were apparently enhanced with contrast. The area expressed with streak-like high intensity on CT images showed no signal on MRI images, which might be considered as vascular void effect (**Figures 2C–F**). From the DCE-MR imaging analysis, we identified the Time Intensity Curve (TIC) of these 2 types of IMHs were quick wash-in and slow washout (**Figure 2G**), which further suggested that capillary and mixed type of IMHs belong to rich blood supply lesions.

**Figure 3:**
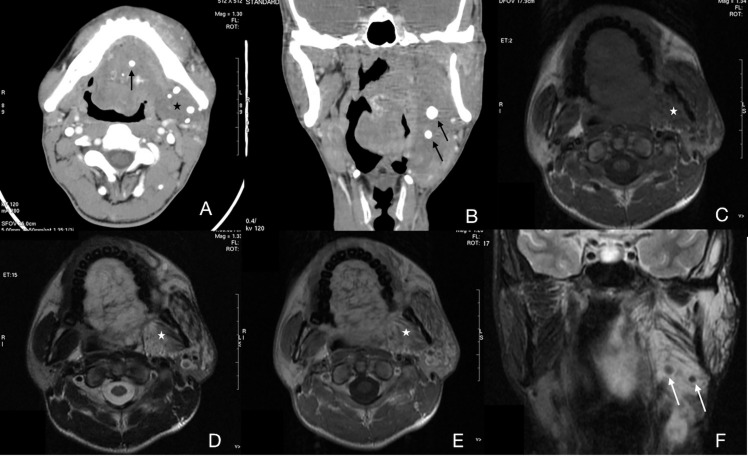
Case 13 (**Supplementary Table 1**), cavernous IMHs. **(A)** Axial CT image showing multiple phleboliths (arrow) in a diffuse, hypo attenuated lesion (star). Oral floor and left masticatory muscles were infiltrated. **(B)** Coronal CT image showing multiple phleboliths (arrow). **(C)** Axial T1WI showing a diffuse, intermediate signal-intensity lesion with a vague border (star). **(D)** Axial T2WI showing a lesion with slightly high signal intensity and a blurred border. **(E)** Axial enhanced T1WI showing no enhancement of the lesion (star). **(F)** Coronal enhanced fat-suppressed T1WI showing multiple round, low signal-intensity structures which indicated phleboliths (arrow).

**Figure 4:**
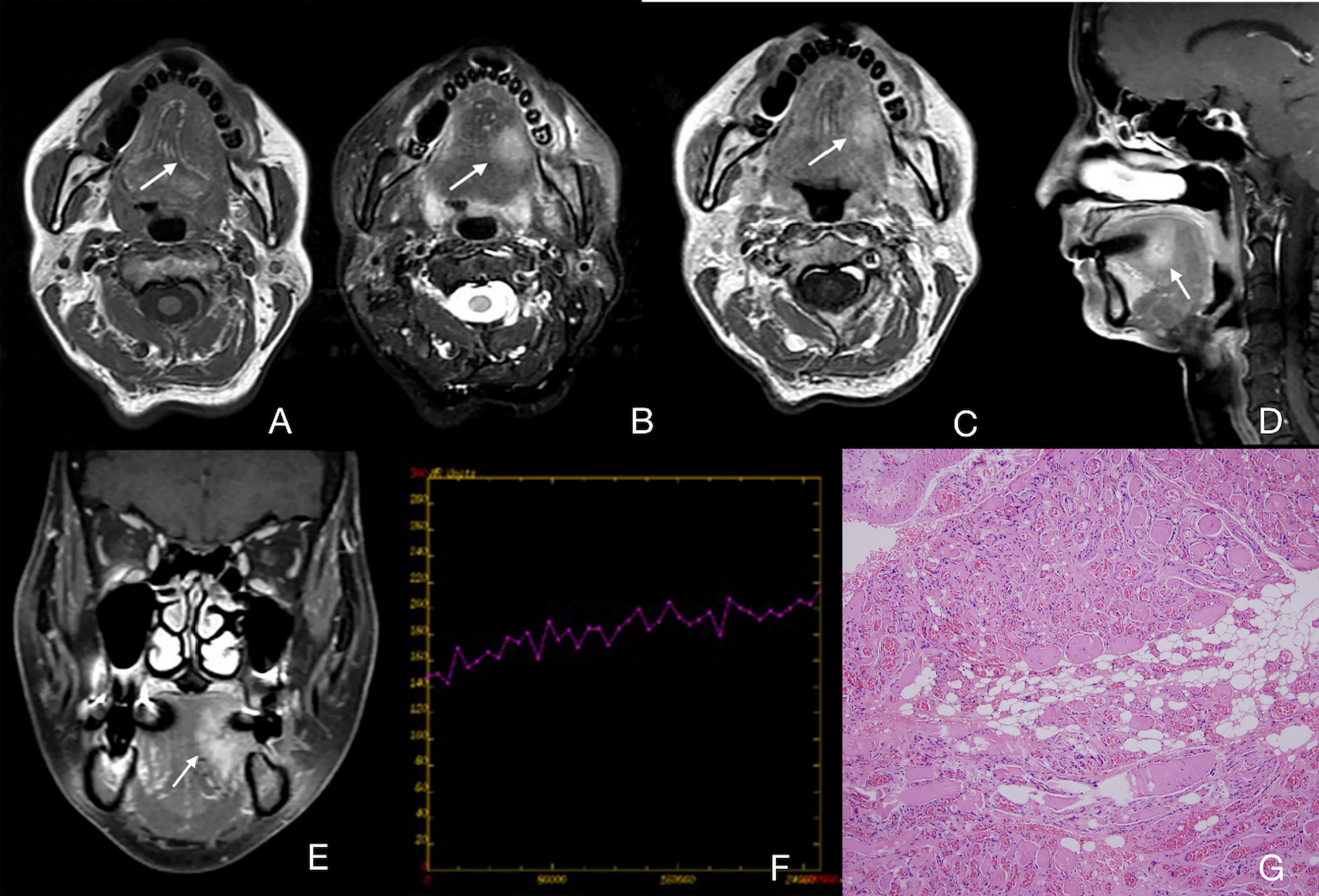
Case 29 (**Supplementary Table 1**), cavernous IMHs. **(A)** Axial T1WI showing an intermediate signal intensity lesion in the left part of tongue with a vague border (arrow). **(B)** Axial T2WI showing a lesion with slightly high signal intensity with a blurred border (arrow). **(C)** Axial enhanced T1WI showing a slightly enhanced lesion (arrow). **(D, E)** Sagittal and coronal enhanced fat suppressed T1WI showing a slightly enhanced lesion in the left part of tongue with a blurred border (arrow). **(F)** TIC analysis showing progressive. **(G)** Histopathologic examination reveals blood vessel diameter >140 μm. Hematoxylin and eosin (H&E) staining. Original magnifications, ×100.

The CT features of the cavernous type of IMHs were basically consistent with venous malformations. On non-contrast imaging, the muscle tissues were apparently thicker in the lesion area without clear boundaries to normal muscles. The density of muscles around the lesion area was similar to or slightly lower than that of normal muscles. Most of the lesion images were slightly enhanced with contrast, and a few lesion images were moderately enhanced with delayed scan. No enlarged venous reflux vessel was observed. Scattered circular calcifications were found in the lesion area of 8 patients, which was assessed as phleboliths (**Figure 3A**). The cavernous type of IMHs showed nonhomogeneous, slightly high or high signal intensity on T2WI and intermediate signal intensity on T1WI, both were slightly enhanced with contrast (**Figures 3C–E**, **4A–E**). Some MRI images showed scattered, circular, and non-signal structures (**Figure 3F**), which were confirmed to be phleboliths after comparing to CT images (**Figure 3B**). The TIC of cavernous IMHs all were progressive (**Figure 4F**), and no enlarged venous reflux vessel was observed. Therefore, our findings indicated that the cavernous type of IMHs belong to relatively poor blood supply lesions. Among all, the presence of vermicular vessels was obviously fewer in cavernous type compared to the other 2 types.

**Figure 5:**
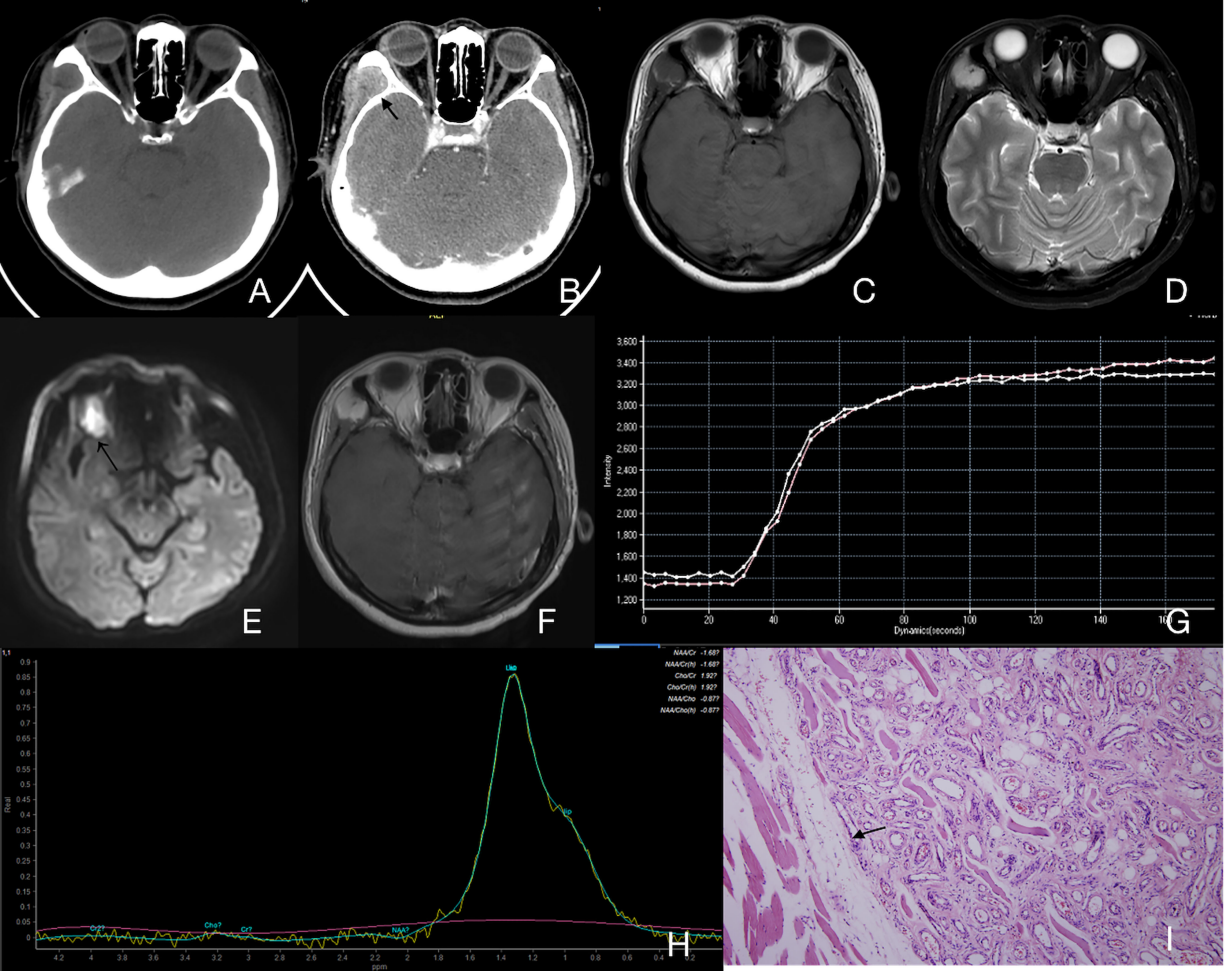
Case 30 (**Supplementary Table 1**), mixed IMHs. **(A)** Axial CT image showing an enlarged right buccal region with uneven attenuation. **(B)** Axial enhanced CT image showing an apparently enhanced mass (arrow). **(C)** Axial T1WI showing a lesion with an intermediate signal intensity and a clear border. **(D)** Axial T2WI showing a lesion with high signal intensity and a clear border. **(E)** The lesion appeared to have a slightly high signal intensity in DWI (arrow), and the average ADC of the lesion was 0.96×10^-3^ mm^2^/s. **(F)** Axial enhanced fat suppressed T1WI showing an unevenly enhanced lesion in the right buccal region. **(G)** TIC analysis showing quick wash-in and slow washout (II). **(H)** MRS showing no Cho peak at 3.2 ppm. **(I)** Histopathology shows that there is a clear boundary between the lesion and the surrounding muscles (arrow). Hematoxylin and eosin (H&E) staining. Original magnifications, ×100.

Misdiagnosis and pathological types—A total of 5 patients (15.6%) were initially diagnosed as sarcoma (4 cases, 12.5%) and granulomatous lesion (1 case, 3.1%). According to pathological classification, the 5 misdiagnosed cases belonged to two types: 3 cases were the cavernous type, 2 cases were the mixed type. Extra functional MRI (fMRI) was performed on these 5 misdiagnosed patients, which indicated that radiologists have tried to distinguish the malignancy of lesions through TIC, ADC or MRS (**Table 4**). The average ADC of the 5 patients was 1.50 × 10^−3^ mm^2^/s. After a comparative study with histopathology, we found that the lack of enlarged venous reflux vessel and the obvious border of mass-like lesions might be the reasons for misdiagnosis (**Figure 5**).

**Table 4:** Extra functional MRI (fMRI) findings of 5 misdiagnosed patients with intramuscular hemangiomas.

Case No*	Site	Initial Diagnosis	Classification	Enhancement Pattern	TIC	ADC	MRS
28	tongue	Granulomatous lesions	cavernous	Progressive	I	1.23	NA
29	tongue	Low-grade mesenchymal tumor	cavernous	Progressive	I	1.55	NA
30	masseter	Low-grade mesenchymal tumor	mixed	Quick wash-in, slow washout	II	0.96	–
31	masseter	Low-grade mesenchymal tumor	cavernous	Progressive	I	1.82	–
32	lip	Hypervascular tumor	mixed	Progressive	I	1.95	NA

NA, not available. *Case number corresponded to **Supplementary Table 1**; ADC value unit was 10^−3^ mm^2^/s.

## Discussion

IMHs consist of vascular and nonvascular components; the latter includes fat, fibers, smooth muscles, etc., with fat as the largest element (13). However, there was no obvious decrease in the signal of fat-saturation sequences of all the observed oral and maxillofacial IMHs patients in this study. This phenomenon was different from the IMHs in other body parts. Pathologically, cavernous type of IMHs accounted for the majority and was expressed similar to venous malformations while the other 2 types were revealed as rich blood supply lesions. Most of the studies suggested that the imaging findings of oral and maxillofacial IMHs are consistent with venous malformations (6, 14–17), and only a few studies indicated that the signal of IMHs lesions can be greatly enhanced (11, 12); however, none of them neither has put forward the concept of rich blood supply status nor combined the enhancement with pathological types of IMHs for analysis. Through the comprehensive analysis of CT and MR images, we found that the capillary and mixed type of IMHs match with the characteristics of rich blood supply lesions, while cavernous IMHs were consistent with previous study results as relatively poor blood supply lesions.

Although there were recurrences in 4 IMHs patients, it is difficult to clearly explain the relationship between recurrence and pathological types, morphological features, or treatment. Even with the confirmation of the lesion boundary by MRI, it was still difficult to remove the lesion completely. Currently we are working on the improvement of IMHs treatments. Distinct from the traditional method, the new treatment confirmed both lesion boundary and blood supply status, through MRI. Patients with a rich blood supply received TAE then flap operation to expose the lesion, followed by electrocoagulation to cause malformed vascular fibrosis. Contrarily, patients with a poor blood supply were directly injected with sclerosing agents if the lesions are small. For diffuse lesions, electrocoagulation can be performed without TAE. This treatment method can reduce the recurrence rate due to incomplete resection and retain as much of the soft tissue as possible. Therefore, an accurate preoperative evaluation of the blood supply and lesion boundary of oral and maxillofacial IMHs is of great value for guiding clinical treatment (18).

This study showed that all pathological types of IMHs mainly yielded an intermediate signal intensity on T1WI, and most of the patients had slightly hyperintense on T2WI (only 2 cases of cavernous IMHs were hyperintense). This phenomenon might be due to the larger vessel diameter in cavernous IMHs causing larger amount of free fluid in blood vessels. On the contrary, smaller vessel cavities were observed in capillary type of IMHs, which might be associated with the slightly high signal intensity on T2WI.

IMHs may involve a single muscle (localized) as well as multiple muscles including masticatory and expression muscles (diffuse). We identified that localized oral and maxillofacial IMHs were more likely to onset at tongue and masseter. The 5 misdiagnosed cases were all localized IMHs patients. The reason for the misdiagnosis may be because diffuse IMHs morphologically have larger difference from tumors, which are easier to be diagnosed; localized IMHs have clearer boundary of lesions with intermediate intensity on T1WI, slightly high intensity on T2WI, and even clearer with contrast, which makes it more likely to be confused with mesenchymal tumors. Extra functional MRI (fMRI) was performed on these 5 misdiagnosed patients, which indicated that imaging doctors have tried to distinguish the malignancy of lesions through TIC, ADC or MRS. Although the average ADC of the 5 patients was 1.50 × 10^−3^ mm^2^/s, of 3 cases were not high enough for distinguishing between localized IMHs or low-grade malignant tumors. TIC results showed that 4 cases were type I, and 1 case was type II. Unfortunately, considering the variance of blood supply in different IMHs, TIC was not able to provide stable diagnostic reference. Only two misdiagnosed patient has received MRS, and Cho peaks were not observed in both cases. Obvious Cho peaks usually can be seen in sarcomas at the site of 3.2 ppm of the H spectrum (19). Therefore, we suggested that the presence of Cho peaks in MRS might be an important criterion for differentiating localized IMHs from low-grade malignant mesenchymal tumors in the maxillofacial region. We need further support from a large number of clinical trials.

Among the 4 patients with embolization before resection, 3 had vermicular vessels in the lesions; however, among the 28 patients who received resection directly without embolization, only 7 (25%) had vermicular vessels in the lesions. We inferred that surgeons tended to determine whether preoperative embolization was necessary based on the presence of vermicular vessels. The percentage of having vermicular vessels was apparently different among the 3 IMHs types, which were 17.6, 33.3, and 66.7% in cavernous IMHs, capillary IMHs, and mixed IMHs cases, respectively (**Table 1**). This observation suggested that the probability of observing vermicular vessels was higher in capillary and mixed IMHs images than that of in cavernous IMHs images. However, it is still too risky to use presence of vermicular vessels alone as a judgement for non-cavernous IMHs since it was also identified in 3 cavernous IMHs cases in this study. No obvious difference was found in the presence of phleboliths among 3 pathological types in this study, except for cavernous IMHs cases. Two mixed IMHs cases had phleboliths as well. The fMRI might be more reliable than morphological features such as presence of vermicular vessels or phleboliths for determining the blood supply status of IMHs. Combined evaluation of presence of vermicular vessels and fMRI might be more accurately for determining the IMHs.

Our findings were inherently restricted to small sample size due to the rarity of the maxillofacial IMHs, we only performed a meaningful analysis on the fMRI analysis in early stage. Although we need prospective surgical pathologic analysis to confirm these findings, it still provides useful clues for unveiling the clinical mechanism of IMHs. From the imaging and pathological findings of IMHs, we suggested that MRS and TIC might be able to provide more information on diagnosis of IMHs and determination of blood supply status.

In conclusion, multi-muscular diffuse IMHs are relatively easy to be diagnosed, and single muscular localized IMHs, with the lack of enlarged venous reflux vessel and the obvious border of mass-like lesions, are tended to be confused with low-grade malignant mesenchymal tumors. Presence of Cho peaks in MRS can be used as a potential marker for differential diagnosis. Treatment of IMHs is highly related to its blood supply status, and simply uses the presence of vermicular vessels for determining blood supply status seem to be far from enough. TIC may be used as criteria for identifying IMHs with rich blood supply. Understanding the morphological and functional imaging features will be a key role in determining the clinical treatment plan for patients with IMHs.

## Data Availability Statement

The original contributions presented in the study are included in the article/**Supplementary Material**. Further inquiries can be directed to the corresponding authors.

## Ethics Statement

The studies involving human participants were reviewed and approved by the 9th People’s Hospital affiliated to Shanghai Jiao Tong university School of Medicine. Written informed consent to participate in this study was provided by the participants’ legal guardian/next of kin. Written informed consent was obtained from the individual(s), and minor(s)’ legal guardian/next of kin, for the publication of any potentially identifiable images or data included in this article.

## Author Contributions

DZ and JW drafted the work or revised it critically for important intellectual content. CZ provided pathological information. XT is responsible for the final approval of the version to be published. LZ is responsible for the substantial contributions to the conception or design of the work. All authors contributed to the article and approved the submitted version.

## Funding

This study has received funding by the research of the “Clinical +Plan” Project of the Shanghai Ninth People’s Hospital, Shanghai JiaoTong University School of Medicine (JYLJ201918), and the Technology Transfer Project of Shanghai Jiao Tong University School of Medicine (ZT202108).

## Conflict of Interest

The authors declare that the research was conducted in the absence of any commercial or financial relationships that could be construed as a potential conflict of interest.

## Publisher’s Note

All claims expressed in this article are solely those of the authors and do not necessarily represent those of their affiliated organizations, or those of the publisher, the editors and the reviewers. Any product that may be evaluated in this article, or claim that may be made by its manufacturer, is not guaranteed or endorsed by the publisher.

## Supplementary Material

Supplementary Figure 1 Case 28 (Supplementary Table 1), cavernous IMHs. **(A)** Axial T1WI showing an intermediate signal intensity lesion in the left part of tongue with a vague border (arrow). **(B)** Axial T2WI showing a lesion with slightly high signal intensity with a blurred border (arrow). **(C)** Axial enhanced T1WI showing a slightly enhanced lesion (arrow). **(D, E)** Coronal and sagittal enhanced fat suppressed T1WI showing a slightly enhanced lesion in the left part of tongue. **(F)** TIC analysis showing quick wash-in and slow washout (II). **(G)** Histopathologic examination reveals that there is no boundary between the lesion and the surrounding muscles. Hematoxylin and eosin (H&E) staining. Original magnifications, ×100.
