# Supplementary figures and images for: Imaging Findings From Different Pathological Types of Oral and Maxillofacial Intramuscular Hemangiomas for Selecting Optimum Management
Source: Front Oncol. 2022 Jan 31;11:792554. doi: 10.3389/fonc.2021.792554 (PMC8841851; doi:10.3389/fonc.2021.792554)

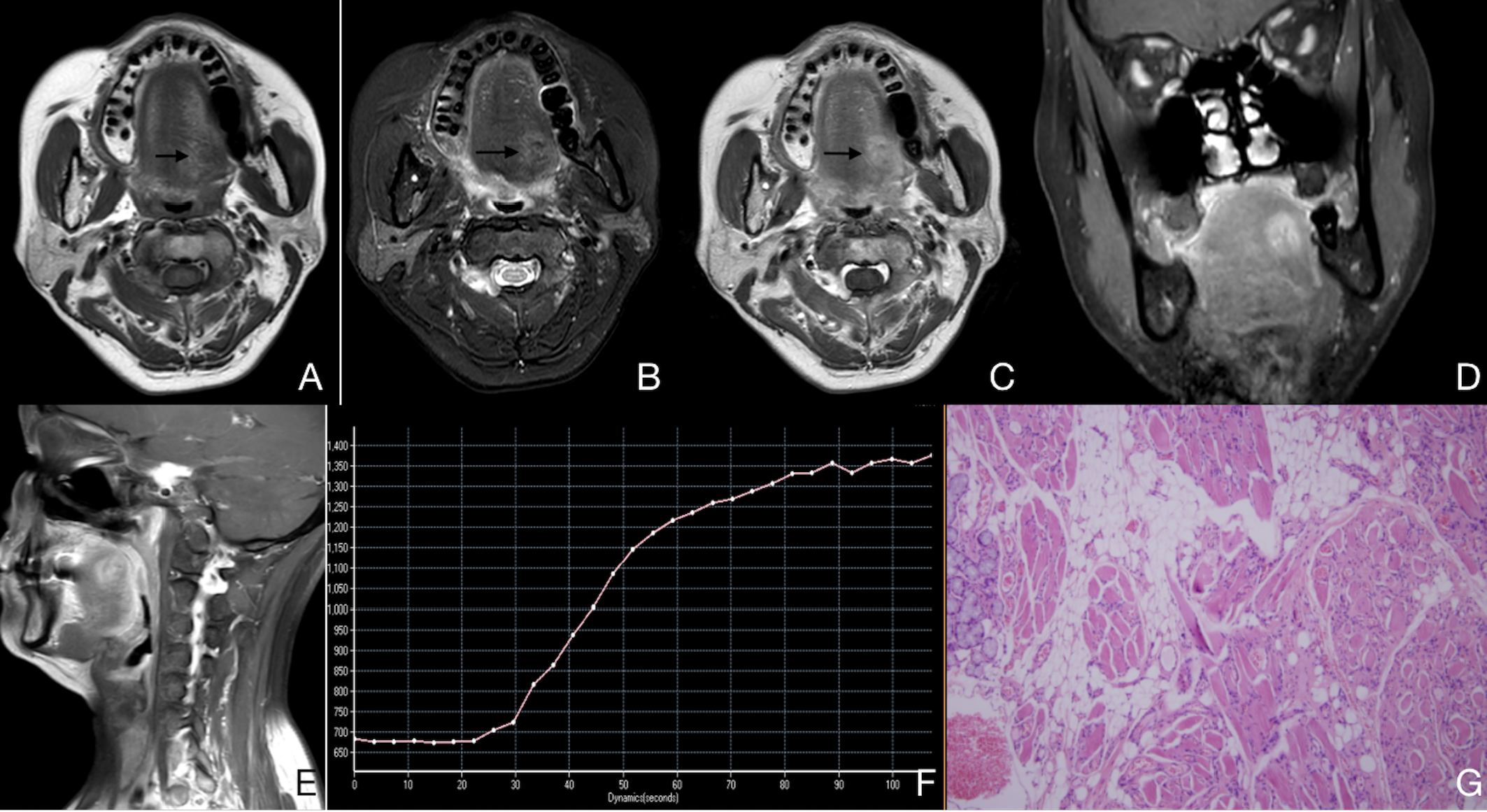

Supplement: Supplementary Figure 1 — Case 28 (Supplementary Table 1), cavernous IMHs. (A) Axial T1WI showing an intermediate signal intensity lesion in the left part of tongue with a vague border (arrow). (B) Axial T2WI showing a lesion with slightly high signal intensity with a blurred border (arrow). (C) Axial enhanced T1WI showing a slightly enhanced lesion (arrow). (D, E) Coronal and sagittal enhanced fat suppressed T1WI showing a slightly enhanced lesion in the left part of tongue. (F) TIC analysis showing quick wash-in and slow washout (II). (G) Histopathologic examination reveals that there is no boundary between the lesion and the surrounding muscles. Hematoxylin and eosin (H&E) staining. Original magnifications, ×100. [file Image_1.tif]
